# 

**Published:** 1860-02

**Authors:** 


### CONTENTS.

ORIGINAL ESSAYS AND COMMUNICATIONS.

The Physiology and Pathology of the Mouth.—The Glands and their Secretions,........ 345
Another Screw Loose,........ 352
Difficult Dentition,........ 357
A Case of Osseous Tumor, with a Tooth imbeded in the part,........ 358

PROCEEDINGS OF SOCIETIES.

Indiana State Dental Association,........ 359
Discussions of the Indiana State Dental Association,........ 364
Meeting of the Michigan Dental Association,........ 374
Constitution and By-Laws of the Cincinnati Dental Association,........ 377

CORRESPONDENCE.

A Letter from Philadelphia,........ 380
Curiosities,........ 382
Subjects for Discussion at the next meeting of the Mississippi Valley Association........ 388

EDITORIAL.

A Correction,........ 388
Associations—Meetings........ 389
Dental Engagement Book,........ 392
The Commencement Exercises,........ 392
The Dentist's Shooting Match........ 392
Teeth........ 391
Removal........ 391

SELECTIONS.

Micrometer giving without calculation the dimensions of Microscopic objects........ 384
False Teeth in Œsophagus fifteen days,........ 385

